# Supplementary material for: Diversity, Distribution, and Biogeography of Freshwater Fishes in Guangxi, China
Source: Animals (Basel). 2022 Jun 24;12(13):1626. doi: 10.3390/ani12131626 (PMC9264779; doi:10.3390/ani12131626)
Supplement: Supplementary file 1 [file animals-12-01626-s001.zip › animals-1744585-supplementary.pdf]

Supplementary Table S1. List of freshwater fishes in Guangxi

Order	Family	Valid	Synonym	Xijiang River;								Rivers flowing into the sea		Yangtze River	Red River
				XR	YYR	HR	LGR	LR	HSR	YR	ZR	NLR	OR	XZR	BDR
Myliobatiformes	Dasyatidae	*Hemitrygon akajei* (Muller & Henle, 1841)[1]	*Dasyatis akajei*	+	+			+		+	+				
Acipenseriformes	Acipenseridae	*Acipenser sinensis* Gray, 1835[1] (CR)		+				+							
Clupeiformes	Clupeidae	*Tenualosa reevesii* Richardson, 1846[1] (CR)		+											
		Clupanodon thrissa (Linnaeus, 1758)[2]										+	+		
		Konosirus punctatus (Temminck & Schlegel, 1846)[2]										+	+		
	Engraulidae	*Coilia grayi* Richardson, 1844[1]		+	+										
Anguilliformes	Anguillidae	*Anguilla japonica* Temminck & Schlegel, 1846[1] (EN)		+	+		+	+	+	+					
		Anguilla marmorata Quoy & Gaimard, 1824[1] (EN)		+				+	+						+
Osmeriformes	Plecoglossidae	*Plecoglossus altivelis* Temminck & Schlegel, 1846[1] (EN)											+		
	Salangidae	*Neosalanx taihuensis* Chen, 1956[3]			+		+		+		+				
		Salanx chinensis (Osbeck, 1765)[1]	*Leucosoma chinensi*	+	+		+			+		+			
		Salanx cuvieri Valenciennes, 1817[2]										+	+		
Characiformes	Serrasalmidae	*Piaractus brachypomus* Cuvier, 1817[3]	*Colossoma brachypomus*		+			+	+						
	Prochilodontidae	*Prochilodus lineatus* (Valenciennes, 1837)[3]		+	+			+	+	+	+				
Cypriniformes	Catostomidae	*Myxocyprinus asiaticus* (Bleeker, 1864)[3] (CR)			+										
	Nemacheilidae	* *Paranemachilus genilepis* Zhu, 1983									+				

* *Paranemachilus pingguoensis* Gan, 2013								+					
* *Paranemachilus jinxiensis* (Zhu, Du & Chen, 2009)	*Yunnanilus jinxiensis*								+				
Traccatichthys pulcher (Nichols & Pope, 1927)	*Micronemacheilus pulcher*	+	+	+	+	+	+	+	+	+	+	+	
* *Traccatichthys taeniatus* (Pellegrin & Chevey, 1936)													+
* *Micronemacheilus pulcherrimus* (Yang, Chen & Lan, 2004)	*Yunnanilus pulcherrimus*						+						
* *Heminoemacheilus parva* Zhu & Zhu, 2014									+				
* *Heminoemacheilus longibarbatus* (Gan, Chen & Yang, 2007)	*Yunnanilus longibarbatus*						+						
* *Heminoemacheilus bailianensis* (Yang, 2013)	*Yunnanilus bailianensis*					+							
* *Heminoemacheilus hyalinus* Lan, Yang & Chen, 1996(EN)							+						
* *Heminoemacheilus zhengbaoshani* Zhu & Chao, 1987							+						
**Yunnanilus retrodorsalis* (Lan, Yang & Chen, 1995)	*Oreonectes retrodorsalis*						+						
* *Oreonectes polystigmus* Du, Chen & Yang, 2008				+	+								
* *Oreonectes luochengensis* Yang, Wu, Wei & Yang, 2011					+								
* *Oreonectes guananensis* Yang, Wei, Lan & Yang, 2011						+							
Oreonectes platycephalus Günther, 1868					+	+	+					+	
* *Oreonectes anophthalmus* Zheng, 1981(EN)								+					
* *Oreonectes guilinensis* Huang, Yang, Wu & Zhao, 2020					+								
Schistura incerta (Nichols, 1931)					+	+	+	+				+	
Schistura fasciolatus (Nichols & Pope, 1927)		+	+	+	+	+	+	+	+	+	+	+	+
* *Schistura paraxena* Endruweit, 2017						+							
* *Schistura alboguttata* Cao & Pope, 2018								+					

* *Triplophysa longipectoralis* Zheng, Du, Chen & Yang, 2009		+		
* *Triplophysa huapingensis* Zheng, Yang & Chen, 2012			+	
* *Triplophysa macrocephala* Yang, Wu & Yang, 2012		+		
* *Triplophysa langpingensis* Yang, 2013			+	
* *Triplophysa fengshanensis* Lan, 2013			+	
* *Triplophysa tianlinensis* Li, Li, Lan & Du, 2016			+	
* *Triplophysa tianlinensis* Li, Lan, Chen & Du, 2017			+	
* *Triplophysa flavicorpus* Yang, Chen & Lan, 2004			+	
* *Triplophysa nandanensis* Lan, Yang & Chen, 1995			+	
* *Triplophysa tianeensis* Chen, Cui & Yang, 2004(VU)			+	
* *Triplophysa anshuiensis* Wu, Wei, Lan & Du, 2018			+	
* *Troglonectes furcocaudalis* (Zhu & Chao, 1987) (VU)	*Oreonectes furcocaudalis*	+		
* *Troglonectes macrolepis* (Huang, Du, Chen & Yang, 2009)		+		
* *Troglonectes lingyunensis* (Liao, Wang & Luo, 1997) (VU)	*Schistura lingyunensis*			+
* *Troglonectes longibarbatus* (Chen, Yang, Sket & Aljancic, 1998)			+	
* *Troglonectes microphthalmus* (Du, Chen & Yang 2008)		+		
* *Troglonectes translucens* (Zhang, Zhao & Zhang, 2006)			+	
* *Troglonectes donglanensis* (Wu, 2013)			+	
* *Troglonectes duanensis* (Lan, 2013)			+	
* *Troglonectes dongganensis* Yang, 2013		+		
* *Troglonectes huanjiangensis* (Yang, Wu & Lan, 2011)	*Oreonectes elongatus* *Troglonectes elongatus*	+		

	* *Troglonectes acridorsalis* (Lan, 2013)							+					
	* *Troglonectes lihuensis*Yang, Wu & Yang, 2012						+						
	* *Troglonectes barbatus* (Gan, 2013)						+						
	* *Troglonectes hechiensis* Zhao, Liu, Du & Luo, 2021						+						
Botiidae	*Sinibotia robusta* (Wu, 1939)	*Botia robusta*	+	+		+	+	+	+	+		+	
	Sinibotia pulchra (Wu, 1939)	*Botia pulchra*		+	+	+	+	+		+		+	
	Sinibotia zebra (Wu, 1939)	*Leptobotia zebra*				+	+						+
	Parabotia fasciata Dabry de Thiersant, 1872			+		+	+	+	+	+	+		+
	Parabotia banarescui (Nalbant, 1965)					+	+						
	Parabotia maculosus (Wu, 1939)					+	+	+					+
	Parabotia kiangsiensis Liu & Guo, 1986					+							
	* *Parabotia lijiangensis* Chen, 1980					+							
	* *Parabotia brevirostris* Zhu & Zhu, 2012							+					
	* *Parabotia parva* Chen, 1980										+		
	Leptobotia posterodorsalis Lan & Chen, 1992						+						+
	Leptobotia pellegrini Fang, 1936			+		+	+	+		+			
	* *Leptobotia guilinensis* Chen, 1980					+	+						+
	* *Leptobotia punctata* Li, Li & Chen, 2008		+										
Cobitidae	* *Protocobitis typhlops* Yang, Chen & Lan, 1993(EN)							+					
	* *Protocobitis anteroventris* Lan, 2013							+					
	* *Protocobitis polylepis* Zhu, Lü, Yang & Zhang, 2008(VU)								+				
	Cobitis sinensis Sauvage & Dabry de Thiersant, 1874				+	+	+	+	+	+	+	+	+
	Cobitis arenae (Lin, 1934)			+		+	+	+	+	+	+	+	+
	* *Cobitis leptosoma* Chen, Sui, He & Chen, 2015					+							
	* *Cobitis wumingensis* Chen, Sui, He & Chen, 2015								+				
	* *Cobitis xui* Tan, Li, Wu & Yang, 2019									+			

	* *Cobitis australis* Chen, Chen & He, 2013			+											
	* *Bibarba parvoculus* (Wu,Yangand &Xiu, 2015)						+								
	* *Bibarba Bibarba* (Chen & Chen, 2007)							+							
	Misgurnus anguillicaudatus (Cantor, 1842)		+	+	+	+	+	+		+		+	+		+
	Paramisgurnus dabryanus Dabry de Thiersant, 1872				+	+							+		
Cyprinidae	*Parazacco fasciatus* (Koller, 1927)											+			
	Zacco acanthogenys (Boulenger, 1901)	*Zacco platypus*	+		+	+	+	+	+	+	+	+	+		
	Opsariichthys bidens Günther, 1873		+	+	+	+	+	+	+	+	+	+	+		+
	Aphyocypris chinensis Günther, 1868												+		
	* *Aphyocypris pulchrilineata* Zhu, Zhao & Huang, 2013							+							
	* *Aphyocypris arcus* (Lin, 1931)	*Yaoshanicus arcus*				+	+	+							
	Aphyocypris normalis Nichols & Pope, 1927	*Nicholsicypris normalis*								+	+	+			
	Rasbora steineri Nichols & Pope, 1927					+	+	+		+	+	+			
	Tanichthys albonubes Lin, 1932(CR)		+												
	Mylopharyngodon piceus (Richardson, 1846)		+	+	+	+	+	+					+		
	Luciobrama macrocephalus (Lacépède, 1803)(CR)		+	+				+							
	Ctenopharyngodon idella (Valenciennes, 1844)		+	+	+	+	+	+	+	+	+		+		
	Squaliobarbus curriculus (Richardson, 1846)		+	+		+	+	+	+	+	+	+			
	* *Atrilinea macrops* (Lin, 1931)(CR)						+								
	Ochetobius elongatus (Kner, 1867)(CR)		+	+		+	+	+		+					
	Elopichthys bambusa (Richardson, 1845)		+	+			+	+	+	+	+				
	Tinca tinca (Linnaeus, 1758)[3]		+												
	Metzia lineata (Pellegrin, 1907)	*Rasborinus lineatus*	+	+		+	+	+	+	+	+	+			
	Metzia formosae (Oshima, 1920) (VU)	*Rasborinus formosae*	+	+				+		+		+			
	**Metzia parva* Luo, Sullivan, Zhao & Peng, 2015							+							

* *Metzia longinasus* Gan, Lan & Zhang, 2009							+					
Sinibrama macrops (Günther, 1868)				+	+	+			+	+	+	+
Sinibrama melrosei (Nichols & Pope, 1927)			+		+	+	+	+	+	+	+	
* *Pogobrama barbatula* (Luo & Huang, 1995)(EN)											+	
Ancherythroculter lini Luo, 1994		+	+	+	+	+	+		+		+	
Pseudolaubuca sinensis Bleeker, 1864		+	+		+	+	+					
Pseudolaubuca engraulis (Nichols, 1927)		+	+				+	+				
Toxabramis houdemeri Pellegrin, 1932		+	+		+		+	+	+	+	+	
* *Toxabramis hoffmanni* Lin, 1934		+										
Hemiculter leucisculus (Basilewsky, 1855)		+	+	+	+	+	+	+	+	+	+	+
Hemiculterella wui (Wang, 1935)			+		+	+	+					+
Pseudohemiculter dispar (Peters, 1880)		+	+	+	+	+	+	+	+	+	+	+
Pseudohemiculter hainanensis (Boulenger, 1900)					+	+		+	+			
Culter alburnus Basilewsky, 1855		+	+		+	+				+		+
Chanodichthys recurviceps (Richardson, 1846)	*Culter recurviceps*	+	+	+	+	+		+	+	+	+	
Chanodichthys mongolicus (Basilewsky, 1855)	*Culter mongolicus*	+	+			+			+			
Chanodichthys dabryi (Bleeker, 1871)	*Culter dabryi*				+							
Chanodichthys erythropterus (Basilewsky, 1855)	*Cultrichthys erythropterus*		+				+					
Parabramis pekinensis (Basilewsky, 1855)		+	+		+	+	+	+	+	+	+	
Megalobrama terminalis (Richardson, 1846)		+	+			+	+	+	+		+	
Megalobrama amblycephala Yih, 1955[3]			+			+	+					
* *Xenocyprioides parvulus* Chen, 1982(VU)											+	
* *Xenocyprioides carinatus* Chen & Huang, 1985(VU)									+			
Distoechodon tumirostris Peters, 1880					+	+						
Xenocypris argentea Günther, 1868		+	+	+	+	+	+	+	+	+		+

Xenocypris davidi Bleeker, 1871			+		+	+			+	+		+		
Plagiognathops microlepis (Bleeker, 1871)	*Xenocypris microlepis*					+			+			+		
Hypophthalmichthys molitrix Bleeker, 1860		+	+	+	+	+	+	+	+	+				
Aristichthys nobilis(Richardson, 1845)		+	+	+	+	+	+	+	+	+	+			
Hemibarbus labeo (Pallas, 1776)		+	+	+	+	+	+	+	+	+	+	+		
Hemibarbus medius Yue, 1995			+		+	+		+	+	+		+		
Hemibarbus maculatus Bleeker, 1871		+	+	+	+	+	+	+	+	+	+	+		+
Hemibarbus macracanthus Lo, Yao & Chen, 1977		+	+		+			+	+					
Hemibarbus umbrifer (Lin, 1931)					+	+			+					
Hemibarbus longirostris (Regan, 1908)					+			+						
Pseudorasbora parva (Temminck & Schlegel, 1842)		+	+		+	+	+	+	+	+	+	+		+
* *Pseudorasbora elongata* Wu, 1939(VU)					+									
Sarcocheilichthys sinensis Bleeker, 1871					+									
Sarcocheilichthys Parvus Nichols, 1930					+	+	+	+				+		
Sarcocheilichthys kiangsiensis Nichols, 1930					+	+								
Sarcocheilichthys nigripinnis (Günther, 1873)				+	+	+		+		+	+	+		
Squalidus argentatus (Suavage & Dabry, 1874)		+	+	+	+	+	+	+	+	+	+	+		
Squalidus wolterstorffi (Regan, 1908)					+	+			+		+	+		
Squalidus atromaculatus (Nichols & Pope, 1927)					+						+	+		
Pseudogobio vaillanti (Suavage, 1878)						+				+		+		
Pseudogobio guilinensis Yao & Yang, 1977					+	+				+		+		
Abbottina rivularis (Basilewsky, 1855)				+	+	+	+					+		
Microphysogobio chenhsienensis (Fang, 1938)				+	+							+		
Microphysogobio chinssuensis (Nichols, 1926)	*Huigobio chinssuensis*		+		+				+	+	+			+

Microphysogobio fukiensis (Nichols, 1926)					+	+			+		+	+		
Microphysogobio tafangensis (Wang, 1935)					+	+								
* *Microphysogobio zhangi* Huang, Zhao, Chen & Shao, 2017			+		+									
Microphysogobio elongate (Yao & Yang, 1977)		+			+	+	+		+			+		
Microphysogobio kiatingensis (Wu, 1930)					+	+	+	+				+		
Microphysogobio labeoides (Nichols & Pope, 1927)		+				+				+	+			
Microphysogobio tungtingensis (Nichols, 1926)					+	+					+			
Microphysogobio pseudoelongatus Zhao & Zhang, 2001											+			
Platysmacheilus exiguus (Lin, 1932)					+	+		+	+			+		
*Saurogobio dabryi*Bleeker, 1871		+	+		+	+	+	+	+		+	+		
Gobiobotia guilingensis Chen, 1989					+									
Gobiobotia meridionalis Chen & Tsao, 1977			+	+	+	+		+	+			+		
Gobiobotia kolleri Bănărescu and Nalbant, 1966						+	+		+	+				
Acheilognathus macropterus (Bleeker,1871)							+			+				
Acheilognathus barbatus Nichols,1926			+		+	+								
Acheilognathus barbatulus Günther, 1873			+		+	+	+	+	+		+	+		
Acheilognathus tonkinensis (Vaillant, 1892)		+	+	+	+	+	+	+	+	+	+	+		
Acheilognathus meridianus (Wu, 1939)	*Paracheilognathus meridianus*				+	+			+			+		
Rhodeus ocellatus (Kner, 1866)		+	+		+	+	+	+	+	+		+		+
Rhodeus spinalis Oshima,1926									+	+	+			
Rhodeus sinensis (Günther, 1868)	*Rhodeus lighti*				+			+						
Rhodeus fangi (Miao,1934)					+									
Puntius semifasciolatus (Günther, 1868)		+	+		+	+	+	+	+	+	+	+		+
Spinibarbus caldwelli (Nichols, 1925)	*Spinibarbus hollandi*	+		+	+	+	+	+	+	+	+	+		
Spinibarbus denticulatus (Oshima, 1926)		+	+	+	+	+	+	+	+					

Sinocyclocheilus macrolepis (Wang, 1989)			+	+		
* *Sinocyclocheilus guilinensis* Ji, 1985		+				
* *Sinocyclocheilus jii* Zhang & Dai, 1992	+	+				
* *Sinocyclocheilus huanjiangensis* Wu, Gan & Li, 2010			+			
* *Sinocyclocheilus yishanensis* Li & Lan, 1992			+			
* *Sinocyclocheilus brevibarbatus* Zhao, Lan & Zhang, 2009				+		
* *Sinocyclocheilus jiuxuensis* (Li & Lan, 2003)				+		
* *Sinocyclocheilus mashanensis* Wu, Liao & Li, 2010				+		
* *Sinocyclocheilus anophthalmus* Li, 1989				+		
* *Sinocyclocheilus altishoulderus* (Li & Lan, 1992)				+		
* *Sinocyclocheilus lingyunensis* (Li, Xiao & Luo, 2000)(VU)					+	
* *Sinocyclocheilus macrophthalmus* (Zhang & Zhao, 2001)				+		
* *Sinocyclocheilus multipunctatus* (Pellegrin, 1931)			+			
* *Sinocyclocheilus longibarbatus* Wang, 1989			+	+		
* *Sinocyclocheilus donglanensis* Zhao, Watanabe & Zhang, 2006				+		
* *Sinocyclocheilus brevis*(Lan & Chen, 1992)			+			
* *Sinocyclocheilus guangxiensis* Zhou & Li, 1998				+		
* *Sinocyclocheilus tianlinensis* Zhou, Zhang & He, 2004(VU)				+		
* *Sinocyclocheilus anatirostris* Lin & Luo, 1986(VU)				+		
* *Sinocyclocheilus flexuosdorsalis* Zhu & Zhu, 2012				+		
* *Sinocyclocheilus furcodorsalis* Chen, Yang & Lan, 1997(VU)				+		
* *Sinocyclocheilus xunleensis* Lan, Zhao & Zhang, 2004(VU)			+			
* *Sinocyclocheilus jinxiensis* (Zheng, Xiu & Yang, 2013)						+
* *Sinocyclocheilus luolouensis* Lan, 2013					+	
* *Sinocyclocheilus anshuiensis* Gan, Wu, Wei & Yang, 2013				+		
* *Sinocyclocheilus ronganensis* Luo, Huang & Wen, 2016			+			

* *Sinocyclocheilus gracilis* Li & Li, 2014					+								
* *SinocyclocheilusSimengensis* Li,Wu, Li & Lan, 2018							+						
* *Sinocyclocheilus Pingshanensis* Li, Li, Lan & Wu, 2018						+							
* *Sinocyclocheilus huangtianensis* Zhu, Zhu & Lan, 2011				+									
* *Sinocyclocheilus guanyangensis* Chen, Peng & Zhan, 2016					+								
* *Sinocyclocheilus punctatus* Lan & Yang, 2017						+	+						
* *Sinocyclocheilus gracilicaudatus* Zhao & Zhang, 2014							+						
* *Sinocyclocheilus brevifinus* Li, Li & Mayden, 2014				+									
Luciocyprinus langsoni Vaillant, 1904(EN)		+	+		+	+	+	+	+				
Acrossocheilus labiatus (Regan,1908)					+	+						+	
Acrossocheilus parallens (Nichols,1931)					+	+						+	
Acrossocheilus kreyenbergii (Regan, 1908)	*Acrossocheilus hemispinus*	+			+	+	+		+	+		+	
Acrossocheilus iridescens (Nichols & Pope, 1927) (VU)	*Acrossocheilus stenotaeniatus* *Acrossocheilus longipinnis*				+	+	+	+	+		+		
Acrossocheilus beijiangensis Wu & Lin, 1977					+	+							+
Acrossocheilus elongatus (Pellegrin & Chevey, 1934)						+	+	+	+		+		
Acrossocheilus yunnanensis (Regan, 1904)					+		+	+	+				
Acrossocheilus clivosius (Lin, 1935)					+	+							
Onychostoma barbatum (Lin, 1931)					+	+	+				+		
Onychostoma barbatulum (Pellegrin, 1908)					+								
Onychostoma leptura(Boulenger, 1900)								+			+		
Onychostoma sima (Sauvage & Dabry, 1874)						+							
Onychostoma gerlachi (Peters, 1881)		+	+	+	+	+	+	+	+	+	+		+

Onychostoma lini (Wu, 1939)					+	+	+					
Onychostoma ovalis rhomboides (Tang, 1942)			+		+	+	+	+	+			
Onychostoma rarum (Lin, 1933)(VU)			+		+	+	+	+	+			
Tor brevifili s(Peters, 1880)		+			+	+	+	+	+			
Tor zonatus Lin, 1935(VU)		+	+		+	+	+	+	+			
Bangana discognathoides (Nichols & Pope, 1927) (CR)						+	+					
Decorus decora (Peters, 1881) (CR)	*Sinilabeo decorus*		+		+	+	+	+	+			
* *Lanlabeo duanensis* Yao, You & Peng, 2018							+					
* *Zuojiangia jingxiensis* Zheng, You, Yang & Peng, 2018									+			
Altigena wui (Zheng & Chen, 1983) (EN)	*Sinilabeo wui*				+	+	+	+				
Labeo rohita (Hamilton, 1822)[3]			+		+	+	+					
Cirrhinus molitorella (Valenciennes, 1844)		+	+	+	+	+	+	+	+	+	+	
Cirrhinus mrigala (Hamilton, 1822)[3]		+	+									
Osteochilus salsburyi Nichols & Pope, 1927		+	+	+	+	+	+	+	+	+	+	
Rectoris posehensis Lin, 1935		+			+	+	+	+	+			
* *Rectoris longibarbus* Zhu, Zhang & Lan, 2012									+			
* *Sinigarra napoense* Zhang & Zhou, 2012									+			
Pseudocrossocheilus bamaensis (Fang, 1981)						+	+					
* *Pseudocrossocheilus liuchengensis* (Liang, Liu & Wu, 1987)						+	+					
Parasinilabeo assimilis Wu & Yao, 1977 (VU)	*Parasinilabeo longicorpus*	+		+	+	+	+					+
* *Parasinilabeo longiventralis* Huang, Chen & Yang, 2007				+								
* *Parasinilabeo longibarbus* Zhu, Lan & Zhang, 2006				+								
* *Stenorynchoacrum xijiangensis* Huang, Yang & Chen, 2014					+							
Semilabeo notabilis Peters, 1880(EN)		+	+		+	+	+	+	+			
Semilabeo obscurus Lin, 1981(VU)							+	+				

* *Cophecheilus bamen* Zhu, Zhang, Zhang & Han, 2011									+				
* *Cophecheilus brevibarbatus* He, Huang, He & Yang, 2015								+					
Pseudogyrinocheilus prochilus (Sauvage & Dabry de Thiersant, 1874)							+						
* *Prolixicheilus longisulcus* (Zheng, Chen & Yang, 2010)									+				
* *Ptychidio jordani* Myers, 1930 (CR)	*Ptychidio macrops*	+	+		+	+	+	+	+				
* *Ptychidio longibarbus* Chen & Chen, 1989(CR)							+						
* *Hongshuia paoli* Zhang, Qiang & Lan, 2008							+						
* *Hongshuia banmo* Zhang, Qiang & Lan, 2008							+						
* *Hongshuia megalophthalmus* (Chen, Yang & Cui, 2006)	*Sinocrossocheilus megalophthalmus*						+						
Hongshuia microstomatus Wang & Chen, 1989	*Sinocrossocheilus microstomatus*						+						
Garra imberba Garman, 1912/	*Garra pingi pingi*												+
Garra orientalis Nichols, 1925		+	+	+	+	+	+	+	+	+	+		
Discogobio yunnanensis (Regan, 1907)					+		+					+	
Discogobio laticeps Chu, Cui & Zhou, 1993					+		+						
Discogobio multilineatus Cui, Zhou & Lan, 1993							+						
Discogobio tetrabarbatus Lin, 1931		+			+	+	+	+	+			+	
Discocheilus wui (Chen & Lan, 1992)							+						
Procypris merus Lin, 1933(EN)		+	+		+	+	+	+	+				
Cyprinus multitaeniata Pellegrin & Chevey, 1936		+	+		+	+	+	+	+				+
*Cyprinus longzhouensis*Yang & Hwang, 1977(EN)					+				+				
Cyprinus acutidorsalis Wang, 1979					+						+		
Cyprinus rubrofuscus Lacepède, 1803	*Cyprinus carpio*	+	+	+	+	+	+	+	+	+	+	+	
Carassioides cantonensis (Heincke, 1892)		+			+	+			+	+	+	+	

		Carassius auratus(Linnaeus, 1758)		+	+	+	+	+	+	+	+	+	+	+	
	Balitoridae	*Liniparhomaloptera disparis* (Lin, 1934)									+	+	+		
		Liniparhomaloptera qiongzhonqensis Zheng & Chen, 1980									+		+		
		Vanmanenia pingchowensis (Fang, 1935)					+	+	+					+	
		Vanmanenia lineata (Fang, 1935)					+	+		+					
		Vanmanenia xinyiensis Zheng & Chen, 1980					+		+						
		* *Vanmanenia homalocephala* Zhang & Zhao, 2000					+	+							
		Yaoshania pachychilus (Chen, 1980)(VU)	*Protomyzon pachychilus*				+								
		Erromyzon sinensis (Chen, 1980)	*Protomyzon sinensis*				+	+			+		+	+	
		* *Erromyzon damingshanensis* Xiu & Yang, 2017							+						
		**Erromyzon yangi* Neely, Conway & Mayden, 2007					+								
		* *Erromyzon kalotaenia* Yang, Kottelat, Yang & Chen, 2012					+								
		Pseudogastromyzon changtingensis Liang, 1942					+							+	
		Pseudogastromyzon fangi (Nichols, 1931)					+	+							
		* *Paraprotomyzon bamaensis* Tang, 1997(VU)							+						
		Beaufortia pingi (Fang, 1930)							+	+	+				+
		Beaufortia kweichowensis (Fang, 1931)					+	+					+		
		* *Beaufortia cyclica* Chen, 1980					+				+				
		Balitora kwangsiensis (Fang, 1930)	*Sinohomaloptera kwangsiensis*	+			+	+	+		+	+			
		* *Balitora ludongensis* Liu & Chen, 2012									+				
Siluriformes	Siluridae	*Sinogastromyzon wui* Fang, 1930		+			+	+	+		+			+	
		Pterocryptis anomala (Herre, 1933)	*Silurus gilberti* *Silurus cochinchinensis*				+	+	+	+	+		+	+	

	Silurus asotus Linnaeus, 1758		+	+	+	+	+	+	+	+	+	+	+
	Silurus duanensis Hu, Lan & Zhang, 2004					+		+					
	Silurus meridionalis Chen, 1977					+	+			+			+
Clariidae	*Clarias fuscus* (Lacepède, 1803)		+	+	+	+	+	+	+	+		+	+
	Clarias gariepinus (Burchell, 1822)[3]			+			+	+	+				
Cranoglanididae	*Cranoglanis bouderius* (Richardson, 1846) (EN)		+	+		+	+	+	+	+			
Pangasiidae	*Sinopangasius semicultratus* Fang & Chaux, 1949[2]										?	+	
Bagridae	*Tachysurus fulvidraco* Bleeker, 1864	*Pelteobagrus fulvidraco*	+	+	+	+	+			+		+	+
	Tachysurus virgatus (Oshima, 1926)	*Leiocassis virgatus*	+			+	+	+				+	
	Tachysurus argentivittatus (Regan, 1905)	*Leiocassis argentivittatus*	+	+			+						
	Tachysurus intermedius Nichols & Pope, 1927	*Pelteobagrus intermedius*	+	+		+		+		+	+	+	
	Tachysurus vachelli (Richardson, 1846)	*Pelteobagrus vachelli*	+	+	+	+	+	+	+	+			+
	Tachysurus crassilabris (Günther, 1864)	*Leiocassis crassilabris*	+	+	+	+	+	+	+	+	+	+	+
	Tachysurus tenuifurcatus (Nichols, 1931)	*Leiocassis tenuifurcatus*				+							
	Tachysurus eupogon (Boulenger, 1892) (CR)	*Pelteobagrus eupogon*				+							
	Tachysurus kyphus Mai, 1978					+						+	
	Tachysurus gracilis Li, Chen & Chan, 2005	*Pseudobagrus pratti*				+	+						+
	Tachysurus brachyrhabdion	*Pseudobagrus adiposalis*				+							+

		Tachysurus lani Chen, 2010	*Pseudobagrus albomarginatus*				+	+						+		
		Hemibagrus guttatus (Lacepède, 1803)	*Mystus guttatus*	+	+	+	+	+	+	+	+		+	+		+
		Hemibagrus pluriradiatus (Vaillant, 1892)	*Mystus pluriradiatus*										+			+
		Hemibagrus macropterus Bleeker, 1870	*Mystus macropterus*	+			+	+	+			+	+	+		
		Mystus gulio (Hamilton, 1822)											+			
	Sisoridae	*Bagarius yarrelli* (Sykes, 1839)														+
		Glyptothorax sinensis (Regan, 1908)	*Glyptothorax fokiensis*			+	+	+	+	+	+	+	+	+		+
		Pareuchiloglanis longicauda (Yue, 1981)					+		+							
	Amblycipitidae	*Liobagrus anguillicauda* Nichols, 1926						+						+		
		Xiurenbagrus xiurenensis (Yue, 1981)					+	+			+					
		* *Xiurenbagrus gigas* Zhao, Lan & Zhang, 2004							+							
		* *Xiurenbagrus dorsalis* Xiu, Yang & Zheng, 2014				+										
	Ictaluridae	*Ictalurus Punetaus* (Rafinesque, 1818)[3]		+	+	+	+	+	+	+	+					
	Loricariidae	*Pterygoplichthys pardalis* (Castelnau, 1855)[3]		+	+				+	+	+					
Mugiliformes	Mugilidae	*liza haematocheilus* (Temminck & Schlegel, 1845)[2]											+			
Beloniformes	Hemiramphidae	*Hyporhamphus intermedius* (Cantor, 1842)[2]		+												
	Adrianichthyidae	*Oryzias latipes* (Temminck & Schlegel, 1846)			+				+							
		Oryzias pectoralis Roberts, 1998						+								
Cyprinodontiformes	Poeciliidae	*Gambusia affinis* (Baird & Girard, 1853)[3]			+		+	+	+	+	+	+	+	+		+
Synbranchiformes	Synbranchidae	*Monopterus albus* (Zuiew, 1793)		+	+	+	+	+	+	+	+		+	+		
	Mastacembelidae	*Mastacembelus aculeatus* (Bloch, 1786)			+		+	+		+	+		+	+		
		Mastacembelus armatus (Lacepède, 1800)		+	+	+	+	+	+	+	+	+	+	+		
Perciformes	Lateolabracidae	*Lateolabrax japonicus* (Cuvier, 1828)[2]		+			+						+			

	Sinipercidae	*Coreoperca whiteheadi* Boulenger, 1900				+	+	+	+	+	+		+	+
		Siniperca roulei Wu, 1930 (VU)	*Coreosiniperca roulei*				+							
		Siniperca loona (Wu, 1939)					+	+						+
		Siniperca liuzhouensis Zhou, Kong & Zhu, 1987						+						
		Siniperca undulata Fang & Chong, 1932					+	+	+			+		+
		Siniperca scherzeri Steindachner, 1892				+	+	+	+	+	+			+
		Siniperca knerii Garman, 1912		+	+	+		+	+	+	+			+
		Siniperca chuatsi (Basilewsky, 1855)[3]			+									
	Centrarchidae	*Micropterus salmoides* (Lacepède, 1802)[3]			+									
	Cichlidae	*Oreochromis mossambicus* (Peters, 1852)[3]		+	+		+	+	+	+	+			
		Oreochromis niloticus (Linnaeus, 1758)[3]		+	+	+	+	+	+	+	+	+		+
		Oreochromis aureus (Steindachner, 1864)[3]		+	+		+	+	+	+	+			
		Coptodon zillii (Gervais, 1848)[3]		+	+		+	+	+	+	+			
Gobiiformes	Odontobutidae	*Odontobutis sinensis* Wu, Chen & Chong, 2002			+		+	+	+		+		+	+
	Eleotridae	*Hypseleotris compressocephalus* (Chen, 1985)						+					+	+
	Butidae	*Bostrychus sinensis* Lacepède, 1801[2]										+	+	
		Eleotris oxycephala Temminck & Schlegel, 1845		+				+			+	+	+	
		Eleotris melanosome Bleeker, 1853					+					+		
		Philypnus chalmersi Nichols & Pope, 1927					+	+	+	+	+		+	
		Neodontobutis hainanensis (Chen, 1985) (VU)	*Philypnus hainanensis*					+	+					
	Gobiidae	*Mugilogobius myxodermus* (Herre, 1935)		+	+		+	+	+		+			
		Glossogobius giuris(Hamilton, 1822)[2]		+			+					+	+	
		Glossogobius olivaceus (Temminck & Schlegel, 1845)[2]										+	+	

		Rhinogobius giurinus (Rutter, 1897)		+	+	+	+	+	+	+	+	+	+	+
		Rhinogobius duospilus (Herre, 1935)					+	+	+	+	+			+
		Rhinogobius filamentosus (Wu, 1939)					+	+	+		+			+
		Rhinogobius leavelli (Herre, 1935)		+			+	+	+		+			+
		* *Rhinogobius yaoshanensis* (Luo, 1989)(EN)						+						
		* *Rhinogobius parvus* (Luo, 1989)(EN)					+				+			
Anabantiformes	Anabantidae	*Anabas testudineus* (Bloch, 1792)											+	
	Osphronemidae	*Macropodus opercularis* (Linnaeus, 1758)		+	+		+	+	+	+	+	+	+	+
		Macropodus ocellatus Cantor, 1842	*Macropodus chinensis*						+					
	Channidae	*Channa maculate* (Lacepède, 1801)		+	+	+	+	+	+	+	+	+	+	+
		Channa gachua (Hamilton, 1822)			+		+							
		Channa asiatica (Linnaeus, 1758)		+	+	+	+	+	+	+	+	+	+	+
		* *Channa nox* Zhang, Musikasinthorn & Watanabe, 2002										+		
Pleuronectiformes	Cynoglossidae	*Cynoglossus trigrammus* Günther, 1862[2]		+										
Tetraodontiformes	Tetraodontidae	*Takifugu ocellatus* (Linnaeus, 1758)[2]		+				+						

(XR: Xunjiang River; YYR: Yongjiang River and Yujiang River; HR: Hejiang River; LGR: Lijiang River and Guijiang River; LR: Liujiang River; HSR: Hongshui River; YR: Youjiang River; ZR: Zuojiang River; NLR: Nanliujiang River; OR: Other southern rivers flowing to the sea; XZR: Xiangjiang River and Zijiang River; BDR: Baidu River)

*Species endemic to Guangxi

[1]Migratory fish

[2]Estuary fish

[3]Exotic fish

(CR) Critically Endangered fishes in China

(EN) Endangered fishes in China

(VU) Vulnerable fishes in China
